# Avarol Induces Apoptosis in Pancreatic Ductal Adenocarcinoma Cells by Activating PERK–eIF2α–CHOP Signaling

**DOI:** 10.3390/md13042376

**Published:** 2015-04-16

**Authors:** Takushi Namba, Rika Kodama

**Affiliations:** Science Research Center, Kochi University, Kohasu, Oko-cho, Nankoku-shi, Kochi 783-8505, Japan; E-Mail: jm-kodamar@kochi-u.ac.jp

**Keywords:** avarol, ER stress, pancreatic ductal adenocarcinomas, CHOP

## Abstract

Avarol is a sesquiterpenoid hydroquinone with potent cytotoxicity. Although resolving endoplasmic reticulum (ER) stress is essential for intracellular homeostasis, erratic or excessive ER stress can lead to apoptosis. Here, we reported that avarol selectively induces cell death in pancreatic ductal adenocarcinomas (PDAC), which are difficult to treat owing to the availability of few chemotherapeutic agents. Analyses of the molecular mechanisms of avarol-induced apoptosis indicated upregulation of ER stress marker BiP and ER stress-dependent apoptosis inducer CHOP in PDAC cells but not in normal cells, suggesting that avarol selectively induces ER stress responses. We also showed that avarol activated the PERK–eIF2α pathway but did not affect the IRE1 and ATF6 pathways. Moreover, CHOP downregulation was significantly suppressed by avarol-induced apoptosis. Thus, the PERK–eIF2α–CHOP signaling pathway may be a novel molecular mechanism of avarol-induced apoptosis. The present data indicate that avarol has potential as a chemotherapeutic agent for PDAC and induces apoptosis by activating the PERK–eIF2α pathway.

## 1. Introduction

Avarol is a sesquiterpenoid hydroquinone isolated from the marine sponge *Dysidea avara* and has pharmacological properties, including antitumor, antiviral, and anti-inflammatory effects [1,2,3]. Avarol has been shown to have antitumor effects against leukemia and lymphoma and increases the production of intracellular superoxide anions [4]. However, the molecular mechanisms of avarol-induced apoptosis are poorly characterized and its antitumor activities for various cancers are largely unknown.

Pancreatic ductal adenocarcinoma (PDAC) is one of the most lethal malignancies and is the fourth leading cause of cancer-related deaths worldwide. Most patients with PDAC receive chemotherapy because of the lack of early stage detection methods for pancreatic cancer [5,6]. Subsequent treatment with gemcitabine (2′,2′-difuluorodeoxycytidine) is the current standard chemotherapeutic agent used for advanced disease [7,8]. However, the gemcitabine treatment effects are limited by the rapid development of gemcitabine resistance in PDAC. Therefore, the identification of new therapeutic agents is required for the effective treatment of PDAC.

Endoplasmic reticulum (ER), an intracellular organelle, specializes in the proper secretion and folding of proteins. Several stresses, such as metabolic and hypoxic stress, induce ER stress response as well as the unfolded protein response (UPR) [9]. Three types of ER transmembrane proteins are important in the ER stress response: protein kinase R-like ER kinase/pancreatic eIF2 kinase (PERK), protein-kinase and site-specific endoribonuclease (IRE1), and activating transcription factor 6 (ATF6) [10]. ER stress response maintains and restores ER homeostasis by inducing ER chaperones, such as the binding immunoglobulin protein (BiP) that mediates protein refolding [11]. However, irreversible ER stress induces apoptosis to eliminate damaged cells via induction of the C/EBP homologous transcription factor (CHOP), which is a transcription factor involved in downregulating Bcl-2 and activating BAX in response to ER stress [12,13,14]. Cancer cells are constantly under certain levels of ER stress due to conditions, such as hypoxia, low nutrients, and high loads of mutant proteins, and proper ER function is dependent on the UPR that suppresses ER stress-induced apoptosis under these conditions [15]. Thus, UPR is a potential therapeutic target for cancers, and drugs that induce excessive ER stress or inhibit ER stress responses have promising antitumor effects [16].

In this study, we screened marine metabolite compounds that have antitumor effects and demonstrated that avarol selectively induces apoptosis in PDAC cells. Analysis of the molecular mechanisms of avarol-induced apoptosis revealed induction of the ER stress response in PDAC cell lines but not in normal like cells. Moreover, avarol specifically activated the PERK–eIF2α pathway, and the consequent CHOP-dependent BAX activation was essential for avarol-induced apoptosis. Thus, PERK–eIF2α–CHOP signaling was characterized as a novel molecular mechanism of avarol-induced apoptosis, indicating that avarol targets ER stress responses and has potential as a novel chemotherapeutic agent for the treatment of PDAC.

## 2. Results

### 2.1. Avarol Selectively Induces Apoptosis in Pancreatic Cancer Cells

**Figure 1:**
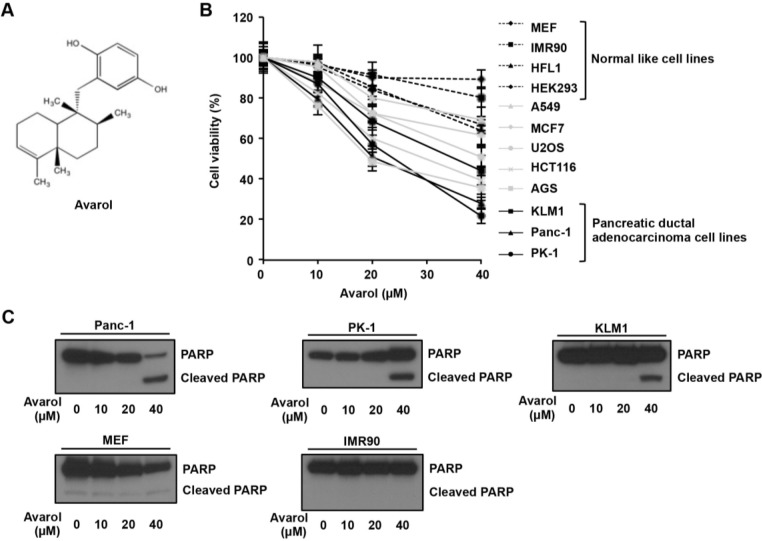
Selective apoptosis induction by avarol in PDAC cells. (**A**) Structure of avarol; (**B**,**C**) Avarol treatment-induced apoptosis in PDAC cell lines but not normal cell lines. MEF, IMR90, HFL1, HEK293, A549, MCF7, U2OS, HCT116, AGS, KLM1, Panc-1, and PK1 cells were incubated with avarol at the indicated concentrations for 24 h. Data are presented as the mean ± standard deviation (SD) of three simultaneously performed experiments; (**B**) Cell viability was determined using MTT assays; (**C**) Panc-1, PK1, KLM1, MEF, and IMR90 cell lysates were subjected to immunoblotting with an anti-PARP antibody. PARP cleavage was used as an apoptosis marker.

Using the cell-based cytotoxicity assay, we performed a marine metabolite screen to identify potential antitumor compounds that selectively induce cancer cell death, MEF (normal like cell), MCF7 (breast cancer cell line), and PK1 (PDCA cell line) were treated with 12 marine metabolites (Supplementary Table S1). Among these, avarol (Figure 1A) was isolated from the marine sponge *D. avara* and was previously shown to have cytotoxic activity against lymphoma and leukemic cells [4]. However, avarol antitumor activity in various cancers has not been investigated, and the ensuing apoptotic molecular mechanisms remain largely unknown. Thus, we examined the effect of avarol on cell viability in several cultured cancer cell lines and normal like cells. All cells were initially treated with avarol at approximately 70% confluence and cell viability was determined using MTT assays. As shown in Figure 1B, avarol selectively induced cell death in PDAC cells (Panc-1, PK1, and KLM1) compared with normal like cells (MEF, IMR90, HFL1, and HEK293). Furthermore, avarol suppressed cell viability in gastrointestinal cancer cell lines AGS and HCT116 and in the osteosarcoma cell line U2OS but not in the lung cancer cell line A549 or MCF7. The cytotoxic activity (IC_50_) of avarol for these cells is also shown in Table 1. To confirm avarol-induced apoptosis in PDAC cells, cleaved PARP was detected as an apoptosis marker. Immunoblotting analyses revealed that 40-μM avarol cleaved PARP in Panc-1, PK1, and KLM1 cells but not in MEF and IMR90 cells (Figure 1C), suggesting that avarol uniquely and selectively induces apoptosis in Panc-1, PK1, and KLM1 cells.

**Table 1:** Cytotoxic effects of avarol for tested cells. IC_50_ were determined by using Figure 1B and Supplementary Figure S1. Data are presented as the mean ± SD of three different experiments.

	IC_50_ values (μM)											
	MEF	IMR90	HFL1	HEK293	A549	MCF7	U2OS	HCT116	AGS	KLM1	Panc-1	PK1
Avarol	>100	>100	78 ± 12	92 ± 9	82 ± 8	70 ± 12	42 ± 7	29 ± 5	19 ± 4	37 ± 9	20 ± 3	23 ± 2

### 2.2. Avarol Induces ER Stress Responses and Upregulates BiP and CHOP Expression

**Figure 2:**
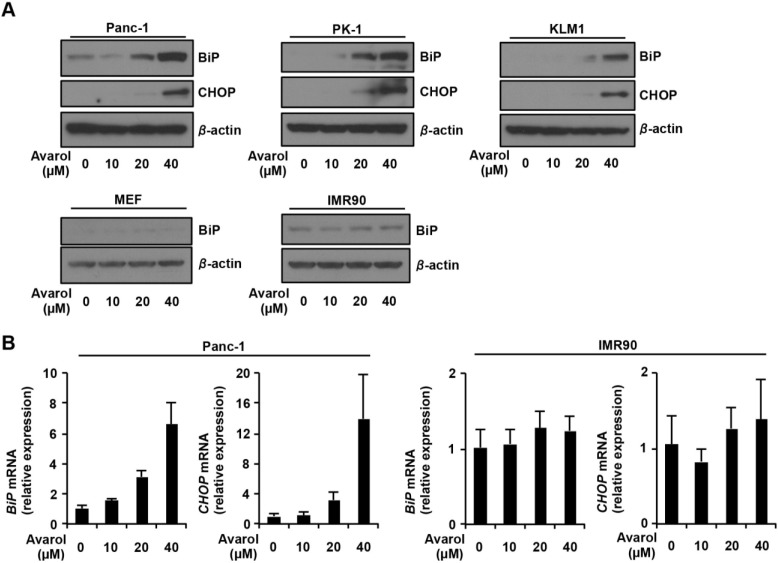
Avarol-induced ER stress in PDAC cells. (**A**) Panc-1, PK1, KLM1, MEF, and IMR90 cells were treated with avarol at the indicated concentrations for 24 h, and whole-cell lysates were subjected to immunoblotting with the indicated antibodies. Blots were excised based on protein sizes; (**B**) Panc-1 and IMR90 cells were treated with avarol at the indicated concentrations for 18 h. Total RNAs were extracted and subjected to qRT-PCR analysis using specific primer sets for *BiP*, *CHOP*, and *GAPDH*, and the data were normalized to *GAPDH* expression. Data are presented as the mean ± SD of triplicate measurements.

Previous studies revealed that avarol increases reactive oxygen species production by inhibiting superoxide dismutase activity, leading to induction of cell death pathways in lymphoma cells [4]. However, the molecular mechanisms of avarol-induced apoptosis remain undetermined. Recently, ER stress response was identified as an important and well-established apoptosis inducing signaling pathway. Thus, we hypothesized that avarol may induced ER stress response in PDCA cells. To test this hypothesis, we analyzed ER stress responses in PDAC cells and normal like cells after treatment with avarol. BiP is an ER chaperon and is also known as an ER stress marker protein [17], whereas CHOP is involved in ER stress response-dependent apoptosis [14]. The expression of these proteins is transcriptionally regulated during ER stress responses, and subsequent upregulation of protein expression has been shown [9]. Immunoblotting analysis showed dose-dependent increases in the expression of BiP and CHOP proteins after treatments with avarol in Panc-1, PK1, and KLM1 cells (Figure 2). However, avarol did not affect BiP protein expression levels in normal like cells at any of the tested concentrations. To investigate mechanisms of BiP and CHOP protein upregulation by avarol, we examined the effects of avarol on *BiP* and *CHOP* mRNA expression using qRT-PCR analyses. These experiments demonstrated dose-dependent increases in *BiP* and *CHOP* mRNA after treatment of Panc-1 cells with avarol. However, avarol treatment did not affect expression of *BiP* and *CHOP* mRNA in IMR90 cells, indicating that avarol specifically induces ER stress responses in PDAC cells via transcriptional activation of BiP and CHOP.

### 2.3. Avarol Activates PERK–eIF2α Signaling Pathway

**Figure 3:**
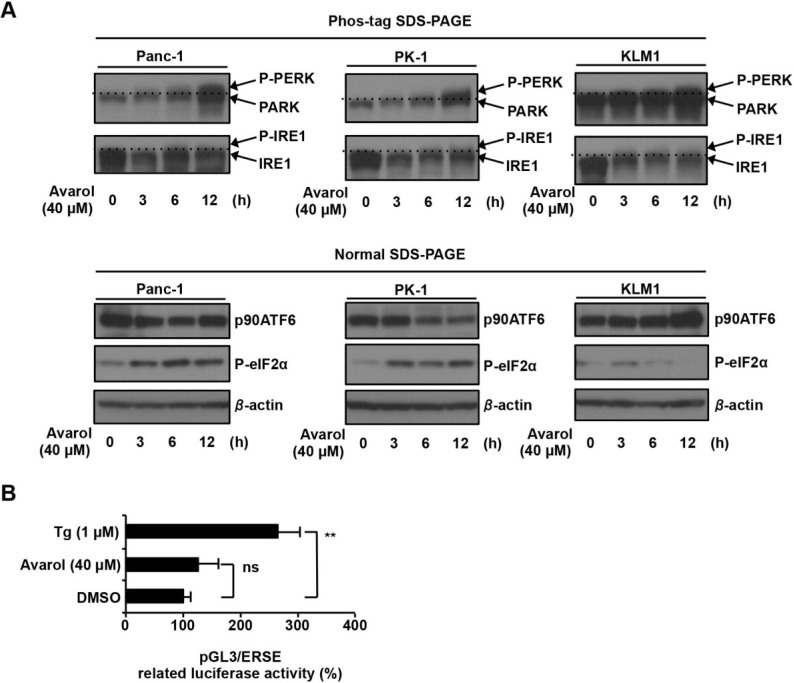
Avarol activates PERK–eIF2α pathway. (**A**) Panc-1, PK1, and KLM1 cells were incubated with avarol (40 μM) for the indicated times. Whole-cell lysates were subjected to immunoblotting with the indicated antibodies using Phos-tag SDS-PAGE or normal SDS-PAGE methods. Blots were cut based on protein sizes and were stripped and re-probed; (**B**) Avarol did not activate ATF6 or IRE1 pathways. Panc1 cells were co-transfected with pRL-SV40 (internal control plasmid carrying the *R. reniformis luciferase* gene) and pGL-3/ERSE and were cultured for 24 h. Cells were treated with Tg, avarol, or DMSO at the indicated concentrations for 8 h, and *P. pyralis* luciferase activity was measured and normalized to *R. reniformis* luciferase activity. Data are presented as the mean ± SD of three simultaneously performed experiments. *P* values were calculated using two-way ANOVA; n.s., not significant; ** *P* < 0.01 (B).

ER stress sensors are known to induce IRE1, ATF6, and PERK–eIF2α signaling pathways. Accordingly, ER stress induced phosphorylation of PERK and IRE1 as well as cleavage of p90ATF6 (inactive form) to p50ATF6 (the active form of the transcriptional factor) and initiated ER stress responses [10]. Thus, to identify signaling pathways that are activated by avarol, we treated Panc-1, PK1, and KLM1 cells with avarol and examined expression as well as phosphorylation of PERK (P-PERK; active form), IRE1 (P-IRE1; active form), and eIF2α (P-eIF2α; active form) and the expression of non-cleaved p90ATF6 (Figure 3). Phos-tag binds phosphorylated proteins and facilitates separation from dephosphorylated proteins on SDS-PAGE gels [18]. Thus, we used Phos-tag SDS-PAGE and immunoblotting methods to separate P-PERK from PERK and P-IRE1 from IRE1. As shown in Figure 3, avarol treatment increased P-PERK but not P-IRE1 protein levels and did not affect expression levels of p90ATF6, indicating that IRE1 and ATF6 pathways are not activated by avarol. Moreover, avarol treatment increased the expression of P-eIF2α, which was phosphorylated by P-PERK. To confirm that avarol did not activate IRE1 and ATF6 pathways, the transcriptional activities of p50ATF6 and X-box protein 1 (XBP1), which are induced by activation of the IRE1 pathway [19], were measured using a reporter plasmid containing ER response element (ERSE) [20]. ERSE has been identified in the promoter of *BiP* genes, and XBP1 and p50ATF6 specifically bind to ERSE to activate transcription [19,21]. Panc-1 cells were transfected with pGL3/ERSE plasmid and were treated with DMSO, avarol, or thapsigargin (Tg), which is an ER stress inducer, and luciferase activity was measured. Tg treatment increased luciferase activity by 2.6-fold compared with DMSO treatment, whereas avarol treatment did not stimulate luciferase activity (Figure 3B). These results confirm that avarol does not affect the ATF6 or IRE1 pathways. Taken together, these observations suggest that avarol specifically activates the PERK–eIF2α pathway under these experimental conditions.

### 2.4. CHOP Is the Key Factor in Avarol-Induced Apoptosis

**Figure 4:**
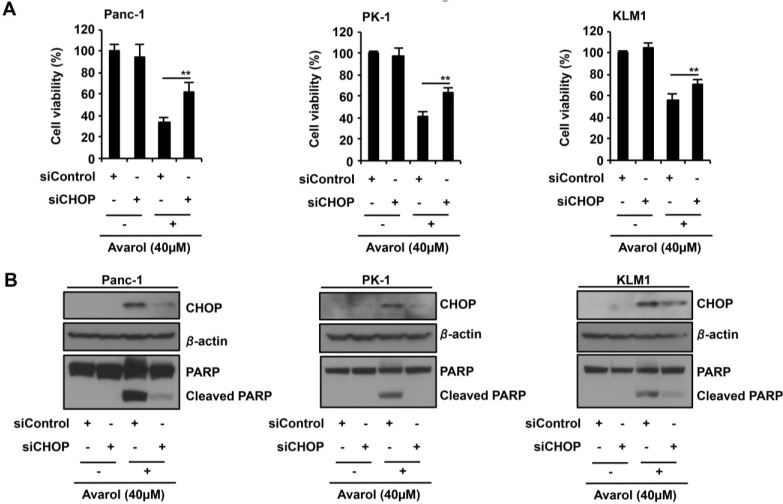

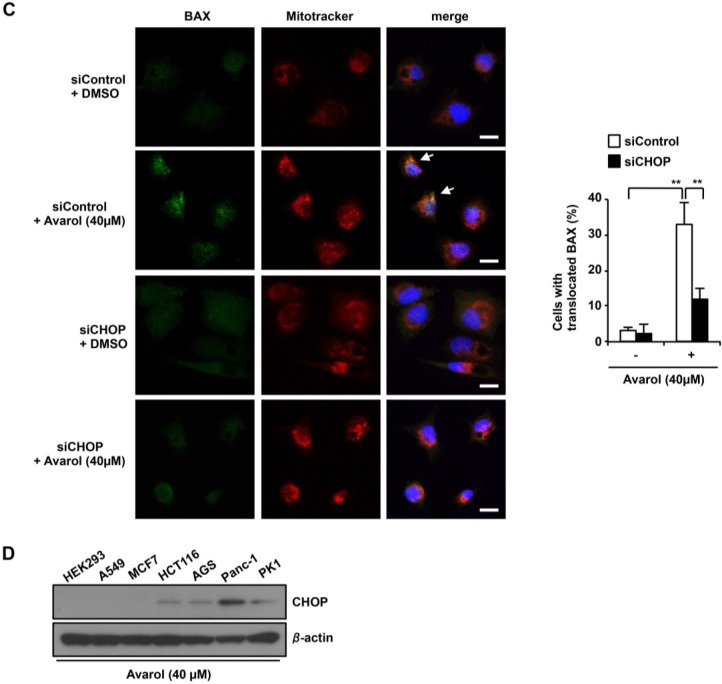
ER stress response-induced CHOP plays a key role in avarol-induced apoptosis. (**A**,**B**) CHOP knockdown suppresses avarol-induced apoptosis; Panc-1, PK1, and KLM1 cells were manipulated with siControl or siCHOP for 24 h and were then treated with 40-μM avarol for 18 h. (**A**) Cell viability was determined using MTT assays. Data are presented as the mean ± SD of three simultaneously performed experiments; (**B**) Whole-cell lysates were subjected to immunoblotting with the indicated antibodies. Blots were cut based on protein sizes; (**C**) The effect of CHOP knockdown on Bax translocation to mitochondria upon avarol treatment. Using the same procedure described in (**A**,**B**), endogenous active Bax was visualized using an anti-Bax antibody (N-20) and confocal microscopy. MitoTracker (red) was used as a mitochondria-specific marker. Merged images are shown, and the yellow color (white arrow) represents co-localization of Bax (green) and MitoTraker (red; scale bar, 20 μm). Percentage BAX translocation was determined by counting three different fields (20–40 cells/field). Data are presented as the mean ± SD of three different experiments. *P* values were calculated using two-way ANOVA; ** *P* < 0.01 (**A**,**C**); (**D**) Avarol induces CHOP expression in Panc-1 and PK1 cells. HEK293, A549, MCF7, HCT116, AGS, Panc-1, and PK1 cells were treated with 40 μM avarol for 24 h, and whole-cell lysates were subjected to immunoblotting with the indicated antibodies. Blots were excised based on protein sizes.

Numerous reports show that CHOP induces apoptosis via the PERK–eIF2α pathway under conditions of ER stress [9]. Because avarol treatment increased CHOP expression in PDAC cells (Figure 2), we investigated whether CHOP induction is essential for avarol-induced apoptosis by transfecting Panc-1, PK1, and KLM1 cells with siRNA against CHOP in the presence or absence of avarol (Figure 4B). Under these conditions, silencing of CHOP expression significantly suppressed avarol-induced cell death and apoptosis (Figure 4A,B). Thus, in further experiments, we examined the effects of the downstream proapoptotic effector Bax on avarol-mediated upregulation of CHOP expression and examined the requirement of CHOP for avarol-induced Bax translocation from the cytosol to the mitochondria [22]. Bax activation was examined in avarol-treated cells using immunofluorescence and confocal microscopy with an antibody that is specific for activated Bax. Following avarol treatment, Panc-1 cells showed punctated mitochondrial staining of active Bax (Figure 4C). In contrast, CHOP knockdown in Panc-1 cells efficiently suppressed avarol-induced Bax accumulation in mitochondria (Figure 4C). Taken together, these observations indicate that avarol-induced CHOP activates BAX and facilitates its translocation from the cytosol to the mitochondria, suggesting that CHOP–BAX signaling is central to avarol-induced apoptosis in PDAC cells. Next, we analyzed whether avarol-dependent induction of CHOP expression is involved in the selectivity of avarol-induced cell death in several types of cell line. We treated HEK293, A549, MCF7, HCT116, AGS, Panc-1, and PK1 cells with 40 μM avarol (Figure 4D). Avarol increased CHOP expression in Panc-1 and PK1 cells, and also slightly induced CHOP expression in HCT116 and AGS cells. However, avarol did not induce CHOP expression in HEK293, A549, and MCF7. The avarol-induced CHOP expression levels are consistent with avarol-induced cell death levels in the tested cell lines (Figure 1B and Figure 4D). In Figure 1B and Figure 2B, we observed similar results, that avarol did not induce CHOP mRNA and cell death in IMR90 cells. Thus, these results suggested that selectivity of avarol-induced cell death might be involved in CHOP induction levels in tested cells.

## 3. Discussion

**Figure 5:**
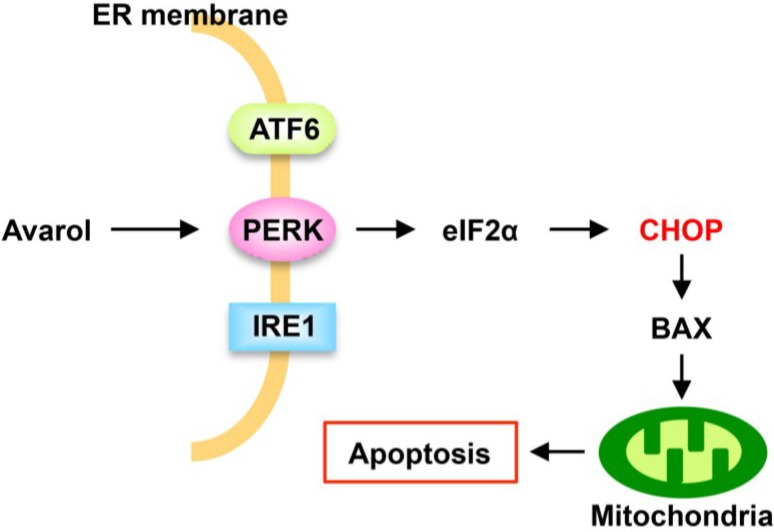
Proposed mechanism of avarol-induced apoptosis.

In the present study, we identified novel mechanisms of avarol and showed that avarol selectively induces apoptosis in PDAC cells. In addition, the pro-apoptotic transcriptional factor CHOP played key roles in avarol-induced apoptosis, indicating that avarol specifically activates the PERK–eIF2α pathway (Figure 5).

In further experiments, we demonstrated that silencing of CHOP using siRNA suppresses avarol-induced apoptotic ER stress responses, corresponding with recent reports that show CHOP as a central signal transducer of ER stress-induced apoptosis [23]. We also showed that avarol activates the PERK–eIF2α pathway, which is a cytotoxic ER stress response pathway that is induced downstream of CHOP [9]. Thus, the present observations of activated PERK–eIF2α–CHOP signaling indicate a fundamental role for avarol in the therapeutic induction of apoptosis signaling. Moreover, avarol did not affect the ATF6 and IRE1 pathways, which are associated with cytoprotective ER stress responses [9], suggesting that avarol is an effective cytotoxic agent that targets ER stress response. In agreement, previous studies show that CO and β-amyloid selectively activate the PERK–eIF2α pathway. However, the molecular mechanisms of this selectivity remain uncharacterized [24,25], warranting further studies to identify primary targets of avarol and to reveal specific mechanisms of PERK–eIF2α pathway activation.

Accumulating evidence suggests that induction of ER stress induces cell death and that solid tumors moderate UPR to accommodate ER stresses, such as hypoxia and low nutrient availability, offering a novel chemotherapeutic target for cancer [15,16]. Thus, excessive ER stress and inhibition of UPR may offer specific targets for inducing apoptosis in cancer cells. Accordingly, several ER-targeted antitumor agents have been examined in clinical and pre-clinical studies, including the proteasome inhibitor bortezomib, cyclooxygenase-2 inhibitor celecoxib, and tyrosine kinase inhibitor sorafenib, which target ER stress responses [26,27,28]. The present data indicated that avarol selectively induces cell death and ER stress responses in PDAC cells but not in normal like cells. In agreement, Muller *et al*. [4] showed that avarol did not induce cell death in several normal cells types. Furthermore, because the pancreas is exposed to strong ER stresses such as low blood vessel-induced hypoxia and protein secretions of digestive enzymes, pancreatic cells are particularly dependent on ER function [29,30]. Therefore, PDAC cells may be particularly sensitive to ER stress-induced cell death by avarol compared with normal cells.

## 4. Materials and Methods

### 4.1. Cell Lines

Human PDAC cell lines Panc-1, PK1, and KLM1, the human fibroblast cells IMR90 and HFL1, the human embryonic kidney cell line HEK293, the human lung cancer cell line A549, the human breast cancer cell line MCF7, the human osteosarcoma cell line U2OS, the human colorectal cancer cell line HCT116, and the human gastric cancer cell line AGS were maintained in DMEM supplemented with 10% FBS, 100-U/mL penicillin, and 100-μg/mL streptomycin. Mouse embryonic fibroblast (MEF) cells were maintained in DMEM supplemented with 10% FBS, 1% NAEE, and 0.5% 2-mercaptoethanol. All cells were maintained at 37 °C in an atmosphere containing 5% CO_2_. PK1, and KLM1 cells were obtained from RIKEN (Ibaraki, Japan). MEF cell was obtained from Dr. Lee (Harvard University, Boston, MA, USA). The other cell lines were obtained from ATCC (Manassas, VA, USA).

### 4.2. Immunoblotting Analysis

Immunoblotting experiments were conducted as previously described [31], and SDS-PAGE was performed using normal polyacrylamide and Phos-tag poly-acrylamide gels (Wako, Tokyo, Japan). Antibodies against P-eIF2α, PERK, IRE1, BiP, CHOP, and PARP were purchased from Cell Signaling Technologies, and those against β-actin and ATF6 were purchased from Sigma (St. Louis, MO, USA) and Santa Cruz (Dallas, TX, USA), respectively. Antibodies were diluted to 1:1000, except for anti-β-actin, which was diluted to 1:10,000. Secondary anti-rabbit and anti-mouse antibodies were purchased from Promega (Madison, WI, USA) and were used at a dilution of 1:5000.

### 4.3. Cell Viability Assays

Cell viability was determined using the 3-(4,5-di-methylthiazol-2-yl)-2,5-diphenyltetrazolium bromide (MTT) method [32]. After treatment with indicated drugs, cells were incubated with MTT solution (1 mg/mL) for 2 h. Isopropanol and HCl were then added to final concentrations of 50% and 20 mM, respectively, and the optical density at 570 nm was determined using a spectrophotometer with a reference wavelength of 630 nm. The IC_50_ values were calculated on the basis of percentage inhibition using the linear regression method.

### 4.4. Real-Time Quantitative PCR and RT-PCR

Real-time quantitative PCR (qRT-PCR) was conducted as previously described [17]. Total RNA was normalized in each reaction using *GAPDH* cDNA as an internal standard. Forward and reverse primers included *BiP*, 5′-TAGCGTATGGTGCTGCTGTC-3′ and 5′-TTTGTCAGGGGTCTTTCACC-3′; *GAPDH*, 5′-CTCAGACACCATGGGGAAGGTGA-3′ and 5′-ATGATCTTGAGGCTGTTGTCATA-3′; and *CHOP*, 5′-TGCCTTTCTCTTCGGACACT-3′ and 5′-TGTGACCTCTGCTGGTTCTG-3′, respectively.

### 4.5. Luciferase Assay

pGL-3/ERSE plasmid was constructed by inserting ERSE (5′-CCAATCAGAAAGTGGCACG-3′) immediately upstream of the *luciferase* gene [20] and was kindly provided by Dr. Gotoh (Kumamoto University, Kumamoto, Japan). Luciferase assays were performed as previously described [32]. Briefly, cells were transfected with 1 μg of *Photinus pyralis* luciferase reporter plasmids (pGL-3/ERSE) and 0.125 μg of the internal standard plasmid bearing the *Renilla reniformis* luciferase reporter (pRL-SV40). *P. pyralis* luciferase activity in cell extracts was then measured using the Dual-Luciferase Assay System (Promega, Madison, WI, USA) and was normalized to *R. reniformis* luciferase activity.

### 4.6. siRNA Experiments

Panc-1, PK1, and KLM1 cells were transfected with a specific siRNA for CHOP (5′-AAGAACCAGCAGAGGUCACAA-3′) [33] and a control siRNA (Santa Cruz, St. Louis, MO, USA) at final concentrations of 50 nM using the X-tremeGENE transfection reagent (Roche, Basel, Schweiz) according to the manufacturer’s instructions.

### 4.7. Immunostaining

Cells were cultured on *poly-l-lysine*-coated coverslips and were fixed in 4% formaldehyde before permeabilization in 0.1% Triton X-100. Immunostaining was performed using polyclonal anti-Bax (N-20, Santa Cruz, St. Louis, MO, USA) as a primary antibody and FITC-conjugated rabbit IgG (Molecular Probes, Carlsbad, CA, USA) as a secondary antibody. Mitochondria were stained using MitoTracker Orange (Molecular Probes, Carlsbad, CA, USA). All images were collected using a confocal microscope and were processed using Adobe Photoshop software (version 13.0.5).

### 4.8. Statistical Analysis

Differences between mean values were evaluated using two-way ANOVA followed by Tukey’s test. Differences were considered significant when *P* < 0.05.

## 5. Conclusions

In conclusion, activation of PERK–eIF2α–CHOP signaling represents an essential mechanism of avarol-induced apoptosis in PDAC cells, suggesting that ER stress responses are a novel therapeutic target for PDAC. Finally, the present data indicated the potential of avarol as a novel chemotherapeutic agent for the treatment of PDAC.

## Supplementary Files

Supplementary File 1

## Abbreviations

ATF6: activating transcription factor 6
BiP: binding immunoglobulin protein
ER: endoplasmic reticulum
ERSE: ER stress response element
CHOP: C/EBP homologous transcription factor
IRE1: protein-kinase and site-specific endoribonuclease
PERK: protein kinase R-like ER kinase/pancreatic eIF2 kinase
PDAC: pancreatic ductal adenocarcinoma
UPR: unfolded protein response
