# Supplementary material for: Avarol Induces Apoptosis in Pancreatic Ductal Adenocarcinoma Cells by Activating PERK–eIF2α–CHOP Signaling
Source: Mar Drugs. 2015 Apr 16;13(4):2376–89. doi: 10.3390/md13042376 (PMC4413216; doi:10.3390/md13042376)
Supplement: Supplementary File 1 [file marinedrugs-13-02376-s001.pdf]

Supplementary Information

Table S1. Cytotoxic effects of various marine metabolites for MEF, MCF7 and PK1 cells. These cells were treated with indicated marine metabolites at the indicated concentrations for 24 h. Data are presented as the average of two independently performed experiments.

	Cell Viability (%)		
	**MEF**	**MCF7**	**PK1**
aaptamine (100 μM)	94	98	91
ara-a (100 μM)	100	93	90
avarol (40 μM)	91	75	25
chitin (20 μg/mL)	100	100	100
chartreusin (100 μM)	48	20	26
K252c (50 μM)	37	25	19
linezolid (100 μM)	100	94	92
manumycin A (40 μM)	74	43	52
phloroglucinol (100 μM)	95	83	92
piericidin (100 μM)	84	74	60
prodigiosin (15 μM)	60	26	28
resistomycin (8 μM)	83	51	48

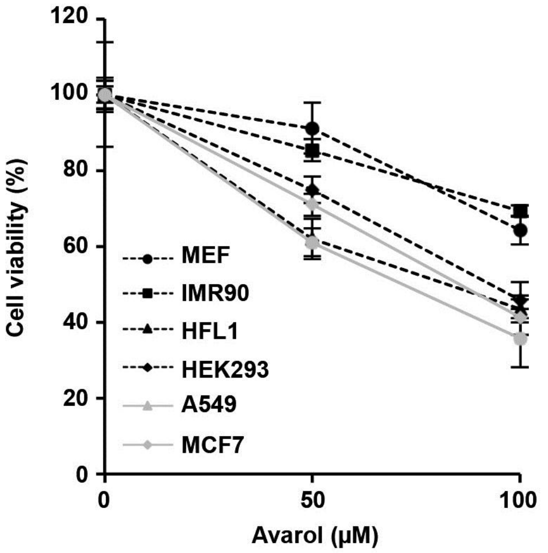

Figure S1. MEF, IMR90, HFL1, HEK293, A549 and MCF7 cells were incubated with avarol at the indicated concentrations for 24 h. Data are presented as the mean ± standard deviation (SD) of three simultaneously performed experiments. Cell viability was determined using MTT assays.

© 2015 by the authors; licensee MDPI, Basel, Switzerland. This article is an open access article distributed under the terms and conditions of the Creative Commons Attribution license (http://creativecommons.org/licenses/by/4.0/).
